# M-CSF Potently Augments RANKL-Induced Resorption Activation in Mature Human Osteoclasts

**DOI:** 10.1371/journal.pone.0021462

**Published:** 2011-06-29

**Authors:** Jason M. Hodge, Fiona M. Collier, Nathan J. Pavlos, Mark A. Kirkland, Geoffrey C. Nicholson

**Affiliations:** 1 The Department of Clinical and Biomedical Sciences, Barwon Health, The University of Melbourne, Geelong, Victoria, Australia; 2 Barwon Biomedical Research, Barwon Health, Geelong, Victoria, Australia; 3 Centre for Orthopaedic Research, School of Surgery, The University of Western Australia, Nedlands, Western Australia, Australia; 4 Rural Clinical School, School of Medicine, The University of Queensland, Toowoomba, Queensland, Australia; Ecole Normale Supérieure de Lyon, France

## Abstract

Macrophage-CSF (M-CSF) is critical for osteoclast (OC) differentiation and is reported to enhance mature OC survival and motility. However, its role in the regulation of bone resorption, the main function of OCs, has not been well characterised. To address this we analysed short-term cultures of fully differentiated OCs derived from human colony forming unit-granulocyte macrophages (CFU-GM). When cultured on dentine, OC survival was enhanced by M-CSF but more effectively by receptor activator of NFκB ligand (RANKL). Resorption was entirely dependent on the presence of RANKL. Co-treatment with M-CSF augmented RANKL-induced resorption in a concentration-dependent manner with a (200–300%) stimulation at 25 ng/mL, an effect observed within 4–6 h. M-CSF co-treatment also increased number of resorption pits and F-actin sealing zones, but not the number of OCs or pit size, indicating stimulation of the proportion of OCs activated. M-CSF facilitated RANKL-induced activation of c-fos and extracellular signal-regulated kinase (ERK) 1/2 phosphorylation, but not NFκB nor nuclear factor of activated T-cells, cytoplasmic-1 (NFATc1). The mitogen-activated protein kinase kinase (MEK) 1 inhibitor PD98059 partially blocked augmentation of resorption by M-CSF. Our results reveal a previously unidentified role of M-CSF as a potent stimulator of mature OC resorbing activity, possibly mediated via ERK upstream of c-fos.

## Introduction

The critical role of macrophage-colony stimulating factor (M-CSF) in osteoclast (OC) differentiation from hematopoietic precursors was demonstrated in mice with osteopetrosis due to disruption of the CSF-1 gene (*Csf1^op^/Csf1^op^*). Osteoclasts were completely absent in these mutant animals [1]–[5]. However, the role of M-CSF in the function of fully-differentiated OCs, in particular bone resorbing activity, is unclear. Two recent studies in mice suggest a role for M-CSF in OC activation. Mice rendered osteopetrotic, either by disruption of the CSF-1 gene (*Csf1^op^/Csf1^op^*), or administration of a neutralizing anti-CSF-1 antibody, displayed this high bone mass phenotype due to total lack, or significant reduction in the number of OCs [6], [7]. Partial correction of osteopetrosis was achieved by either co-expression of cell-surface M-CSF in the case of the *Csf1^op^/Csf1^op^* background, or cessation of neutralizing anti-CSF-1 antibody administration. In both cases there were equivalent levels of OCs to those seen in control animals, leading the authors to deduce that the rate of bone resorption in animals with sub-optimal levels of M-CSF was slower.

In short-term (6 h) semi-pure cultures of isolated neonatal rat OCs, human M-CSF enhanced survival, motility and cytoplasmic spreading, but inhibited resorbing activity, in a concentration-dependent manner; maximum effect at 0.5 ng/mL [8]. In contrast, in 6–18 h cultures of isolated human fetal OCs, M-CSF had no apparent effect on resorbing activity but did enhance survival; maximum effect at 50 ng/mL [9]. Experiments in a human bone marrow osteoclastogenesis assay suggested that resorption per OC was enhanced in the presence of M-CSF 50 ng/mL [10]. Likewise, in an osteoclastogenesis assay employing human colony forming unit-granulocyte macrophage (CFU-GM) precursors, we found that resorption per OC was increased in the presence of M-CSF 25 ng/mL [11]. However, investigation of resorbing activity in osteoclastogenesis assays is problematic because of the confounding effects of proliferation, differentiation and fusion. Furthermore, these findings were inconsistent with the widely held view [12]–[16] that, in fully differentiated OCs, the role of M-CSF is limited to regulation of survival and motility while receptor activator of NFκB ligand (RANKL) and perhaps interleukin-1 (IL-1) [17], [18] are the key regulators of resorption activation.

To address this knowledge gap, we established a mature OC model based on that of Fuller and colleagues [17] that would allow the characterization of the role of M-CSF in the short-term regulation of resorption activation and survival in these cells. We generated OCs *en masse* by treating CFU-GM-derived cells with M-CSF and RANKL for 14d [19]. The OCs were then either detached from the plastic and cultured on dentine slices for 4–72 h for resorption and survival assays, or used *in situ* for transcription factor activation and signalling phosphorylation assays. We demonstrate that although RANKL is necessary for resorption, M-CSF 10–25 ng/mL potently augments RANKL-induced resorption, an effect that is not due to enhanced survival but, rather, due to increased activation of resorption in OCs. We also demonstrate that M-CSF potentiates RANKL-induced c-fos activation and extracellular signal-regulated kinase (ERK) 1/2 phosphorylation, and that the resorption-stimulating effect of M-CSF is blocked by the mitogen-activated protein kinase (MEK) inhibitor PD98059. This is the first study to definitively identify M-CSF as a potent enhancer of resorption activation of mature OCs, a paradigm-shift for its role in physiology and pathophysiology.

## Materials and Methods

### Materials

Eagle's MEM, penicillin/streptomycin, paraformaldehyde, Sigma Cell Dissociation Solution (1X) non-enzymatic (Cat. No. C5914), Fast Garnett GBC, MEK1/2 inhibitor PD98059 and naphthol AS-BI-phosphate were purchased from Sigma. Non-essential amino acids (100X) and FBS were purchased from Bovogen (Melbourne, Australia). Ficoll-Paque was purchased from Pharmacia Biotech. MethoCult GF H4534 (Iscove's MDM containing 1% methylcellulose, 30% FBS, 1% BSA, 10^−4^ M 2-mercaptoethanol, 2 mM L-glutamine, 10 ng/ml recombinant human GM-CSF, 10 ng/ml IL-3, and 50 ng/ml stem cell factor) was purchased from Stem-Cell Technologies. Human M-CSF and neutralising polyclonal antibody against M-CSF was purchased from Chemicon. Primary rabbit monoclonal antibodies raised against β-actin, c-fos and total or phosphorylated forms of IκBα and ERK1/2 and HRP-conjugated anti-rabbit polyclonal antibody were from Cell Signaling Technology (MA, USA). Soluble RANKL coupled to GST fusion protein (RANKL) was generously provided by Drs Matthew Gillespie and Julian Quinn (Prince Henry's Institute, Monash Medical Centre, Melbourne, Australia). Transcription factor activation kits incorporating detection antibody for the p65 subunit of Nuclear Factor kappa B (NFκB) and c-fos component of AP-1 were purchased from Pierce Biotechnology (Illinois, USA). TransAM assay kits for Nuclear factor of activated T-cells, cytoplasmic 1 (NFATc1) were from Active Motif (California, USA).

### Ethics statement

Human umbilical cord blood was obtained with written informed consent from healthy donors under a protocol approved by Barwon Health Human Research and Ethics Committee.

### Mature human osteoclasts

Collection of human umbilical cord blood, isolation of a mononuclear cell fraction, expansion of CFU-GM-derived OC precursors and differentiation of mature human OCs have been previously described [19]. Specifically, a mononuclear cell fraction of cord blood (CBMC) was isolated by Ficoll-Paque density gradient centrifugation. CBMCs (3×10^6^ cells/culture) were suspended in 3.0 ml Methocult GF H4534 in 6-well plates and incubated at 37C in humidified atmosphere of 5% CO^2^-air for 10 days. Pooled colonies (CFU-GM-derived OC precursors) were harvested into PBS. Precursors (6.5×10^6^ cells/175 cm^2^) were then seeded into 175 cm^2^ tissue culture flasks. The cells were cultured in 20 mL of MEM containing 10% FBS, nonessential amino acids, penicillin 50 U/ml, streptomycin 50 µg/ml, 2 mM L-glutamine, M-CSF (25 ng/ml), and RANKL (125 ng/ml) for 14–21 days. The cultures were refreshed weekly by replacing additives in one half volume of media. Mature OCs were used *in situ* for signalling and transcription factor assays or dissociated and re-settled on dentine slices for survival and resorption assays.

### Mature OC survival and resorption assay

Mature OC cultures were treated with dissociation buffer (5 mL/75 cm^2^ flask) for 30 min at 37C when most were detached by agitation. Remaining adherent cells were removed with a cell scraper. Detached OCs were washed, pelleted by centrifugation (200 g; 2 min), resuspended in MEM/10% FBS and settled onto round dentine slices (6 mm diameter) in 96-well plates (n = 500–1000 OCs/well) and cultured for 4–72 h in 200 µL of media. Cells were fixed in 1% formalin and reacted for TRAP as previously described [19]. The formation of OCs was assessed by transmission light microscopy; quantified using microcomputer image analysis software (MCID - Imaging Research Inc. Ontario, Canada). F-actin stained sealing zones were quantified by confocal microscopy using rhodamine-conjugated phalloidin [20]. Cells were removed from dentine slices by brief sonication in chloroform:methanol 2∶1. Xylene-free black ink was applied to the surface of each slice and residual ink removed by wiping on absorbent paper. Resorption was assessed by reflective light microscopy and the percentage area resorbed was quantified using MCID software. This method was validated by comparison with scanning electron microscopy [21].

### NFκB, c-fos and NFATc1 transcription factor activity

Mature OC cultures in 6-well plates were rinsed, pre-treated with or without M-CSF (37.5 ng/mL) for 18 h in MEM/0.2%FBS and then treated with RANKL (125 ng/mL) or vehicle for 30 min. Nuclear extracts (Ne-PER; Pierce, IL, USA) were assessed for activation of NFκB(p65), c-fos and NFATc1 using ELISA-based transcription factor assay kits. Briefly, an oligonucleotide containing the consensus binding sequence for the relevant transcription factor is immobilised to a 96-well plate. Transcription factor contained in nuclear extracts binds specifically to this oligonucleotide and is detected through use of an antibody directed against either p65, in the case of NFκB, c-fos or NFATc1. Addition of a secondary antibody conjugated to horseradish peroxidise provides sensitive chemiluminescent or colorimetric readout that is quantified by spectrophotometry.

### ERK1/2, IκBα signalling and c-fos Western Blot

Mature OCs in 25 cm^2^ flasks were serum-starved for 18 h when media was replaced with MEM/10%FBS with vehicle, RANKL (125 ng/mL), M-CSF (25 ng/mL) or both. Cytoplasmic extracts (M-PER, Pierce, IL, USA) were collected at 0, 5 min and 10 min. For c-fos expression, mature OCs were treated with or without M-CSF for 18 h before cell lysis. Total protein was subjected to SDS-PAGE on 4–20% gradient gels (NuSep, Georgia, USA). Protein gels were transferred to nylon membranes (Amersham Biosciences, Buckinghamshire, UK) overnight at 4C (50 V; 25 mA) and blocked in PBS containing 5%BSA; 0.2% Tween 20 for 1 h. Immunodetection used primary rabbit monoclonal antibodies that recognised either total or phosphorylated forms of IκBα, ERK1/2, c-fos, or β-actin (Cell Signaling Technology, MA, USA). Secondary antibody was HRP-conjugated anti-rabbit polyclonal antibody (Cell Signaling Technology, MA, USA) A chemiluminescent substrate followed by autoradiography was used for detection (GE Healthcare Bio-Sciences, NJ, USA). MCID software was used for densitometric analysis.

### Statistical analyses

Data are expressed as the mean ± SEM where applicable. Differences between groups where determined using one-way ANOVA followed by Fisher's multiple comparison test, two-way ANOVA – general linear model (GLM) followed by Tukey's post-hoc test or unpaired T-test. Statistical significance was set at P<0.05. Treatment groups with annotations containing the same letter are not significantly different. (For example: 3 groups annotated with “a”, “b” and “c”, are all different to each other. In the case of 3 groups annotated with “a”, “ab” and “b”, “a” is different to “b”, whereas “a” and “ab” are not different, likewise “ab” and “b”.)

## Results

### M-CSF acutely augments RANKL-induced resorption in mature human OCs

**Figure 1:**
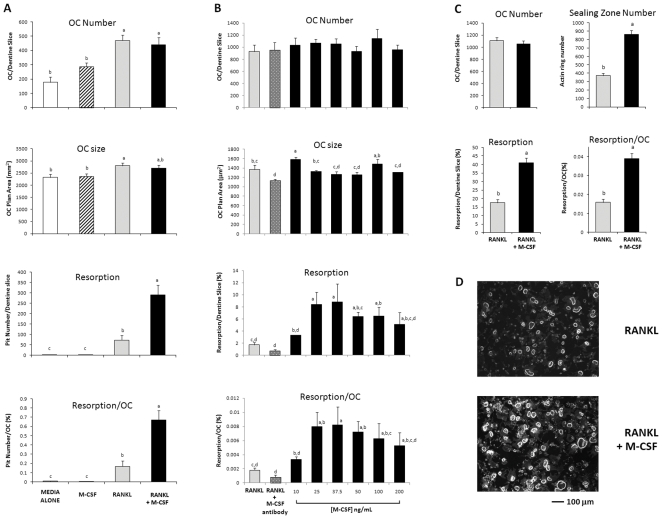
M-CSF augments RANKL-induced resorption and F-actin sealing zone formation by harvested OCs. Mature OCs were formed from CFU-GM precursors cultured on plastic in the presence of RANKL (125 ng/mL) and M-CSF (25 ng/mL) for 21 d. Mature OCs were then detached with non-enzymatic dissociation buffer, re-adhered to dentine slices and cultured for a further 72 h. (A) Survival and activity of harvested OCs cultured in media alone or combinations of RANKL (125 ng/mL) and M-CSF (25 ng/mL); data representative of 6 independent experiments. (B) Concentration-dependent stimulation of RANKL-induced resorption by M-CSF; data representative of 3 independent experiments. RANKL (125 ng/ml) added to all cultures. (C) M-CSF (25 ng/mL) potentiates RANKL-induced (125 ng/mL) sealing zone formation. (D) Photomicrographs show OCs stained for F-actin. Results expressed as mean ± SEM (n = 6 dentine slices/group). Groups with different annotations are significantly different; Panel A&B p = 0.001; one-way ANOVA; Fishers multiple comparison test; Panel C p = 0.001; Two-sample T-test.

We assessed the survival and resorbing activity of mature human OCs by seeding freshly harvested cells onto dentine slices and culturing them for 72 h. Survival of OCs cultured in media/10% FBS alone was enhanced (62% increase) by the addition of M-CSF (25 ng/mL) but more effectively so (165% increase) by RANKL (125 ng/mL). Neither M-CSF nor RANKL had any substantial effect on OC plan area (size). In the absence of RANKL, resorption was negligible. Activation of RANKL-induced resorption per OC was markedly enhanced (300% increase) by the addition of M-CSF (25 ng/mL) (Fig. 1A). In six independent experiments, the addition of M-CSF (25 ng/mL) resulted in a 220±12% (mean±SEM) increase in resorption compared to RANKL (125 ng/mL) alone (p = 0.004, not shown). This effect was concentration dependent (Fig. 1B). At M-CSF concentrations between 50 and 200 ng/mL, a concentration-dependent, although non-significant, trend to decreased resorption was apparent. In cultures treated with RANKL only, co-treatment with a neutralising antibody to M-CSF had no significant effect on survival or resorption, indicating that endogenous M-CSF [11] is not necessary for RANKL-induced survival or resorption activation (Fig. 1B). A 130% stimulation of resorption in the presence of M-CSF (37.5 ng/mL) was accompanied by an equivalent increase in sealing zone formation when compared to treatment with RANKL alone (Figs. 1C and 1D).

**Figure 2:**
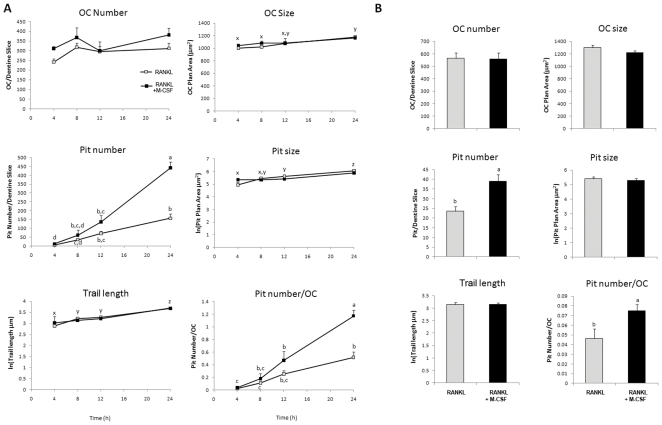
M-CSF rapidly potentiates RANKL-induced resorption by harvested OCs. Mature OCs were re-adhered to dentine slices and cultured for up to 24 h in RANKL (125 ng/mL) alone or RANKL (125 ng/mL) + M-CSF (25 ng/mL). A. Time course of OC survival and resorption. Results expressed as mean ± SEM (n = 6 dentine slices/group). Groups with different annotations (a,b,c) are significantly different for treatment; P<0.05 ANOVA GLM Tukey pairwise comparisons. Groups with different annotations (x,y,z) are significantly different for time; P<0.003 ANOVA GLM Tukey pairwise comparisons; data from one experiment. B. Potentiation of OC activity by M-CSF (25 ng/mL) at 6 h of treatment. Results expressed as mean ± SEM (n = 6 dentine slices/group). P<0.05 Two-sample T-test; data from one experiment.

In our 72 h cultures, we could not exclude the possibility that the observed resorption-stimulating effect was due to ongoing OC differentiation in the presence of M-CSF. In CFU-GM osteoclastogenesis cultures we previously found a linear increase in OC plan area over three weeks due to progressive cell fusion [19] yet in the current study, no substantive increase in OC plan area was observed with M-CSF, suggesting an absence of significant cell fusion. Nevertheless, to exclude this possibility, we conducted experiments over a shorter time of 4–24 h. Since the small amount of dentine resorption present in these short-term assays could not be accurately quantified using whole-slice image analysis, we counted resorption pits at higher magnification and individually measured their plan area and diameter or, in the case of non-circular “trail or trench” pits, trail pit linear length (trail length). In these experiments, OCs were treated with RANKL (125 ng/mL) alone or together with M-CSF (25 ng/mL) (Figs. 2A and 2B). No significant effect on OC number was seen over this period. In the presence of RANKL only, the resorption pit number, size and trail length increased with time in a linear manner. Co-treatment with M-CSF increased the pit number and pits per OC at each time point (200% at 24 h) but had no substantive effect on the pit size or trail length (Fig. 2A). The effect of M-CSF at 6 h in an independent experiment is shown in Fig. 2B. The addition of M-CSF to RANKL had no effect on OC characteristics but increased the number of pits and pits per OC by 66% and 62%, respectively.

### M-CSF pre-treatment facilitates RANKL-induced activation of c-fos but not of NFκB

**Figure 3:**
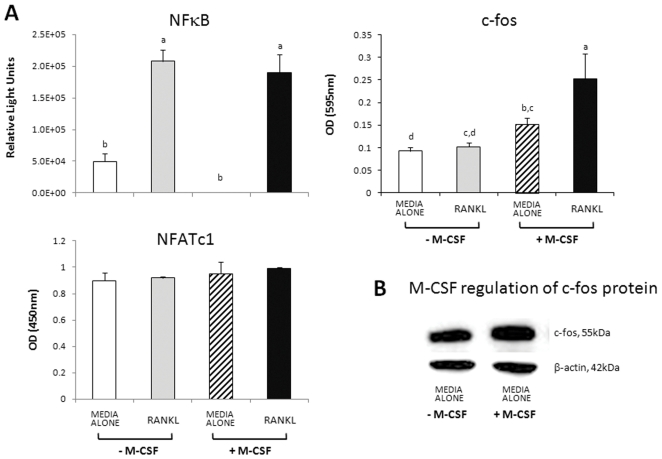
M-CSF pre-treatment facilitates RANKL-induced activation of c-fos but not of NFκB. NFATc1 is not activated by either RANKL or M-CSF. Mature human OCs cultured in 6-well plates for 21 d were serum-starved for 18 h in the absence or presence of M-CSF (37.5 ng/mL) followed by stimulation with RANKL (125 ng/mL) for 30 min. A. Relative activation of transcription factors quantified using ELISA-based transcription factor activation assays: NFκB(p65 subunit) and c-fos (Pierce Biotechnology, USA); NFATc1 (Active Motif, USA); representative data from 2 independent experiments. Results expressed as mean ± SEM (n = 3 wells/group). Groups with different annotations are significantly different; p = 0.001; one-way ANOVA; Fishers multiple comparison test. B. Western analysis of c-fos protein; mature OCs cultured for 18 h in media alone, with or without M-CSF (25 ng/mL).

In mature human OCs, NFATc1 is not activated by either RANKL or M-CSF. Acute treatment with RANKL of OCs that had been serum-starved for 18 h produced a 320% activation of NFκB at 30 min (Fig. 3A). Pre-treatment with M-CSF (37.5 ng/mL) for 18 h had no effect on basal or RANKL-induced activation of NFκB (Fig. 3A). In contrast, in control OCs, RANKL did not activate c-fos, whereas pre-treatment with M-CSF increased basal activation by 70% and RANKL-induced activation by 190% (Fig. 3A). We also found that M-CSF pre-treatment increased basal c-fos protein expression by 41.5% (Fig. 3B). No effect on NFATc1 activation was seen with M-CSF pre-treatment or acute RANKL treatment (Fig. 3A).

### Phosphorylation of ERK1/2 by M-CSF is augmented by co-treatment with RANKL in mature human OCs

**Figure 4:**
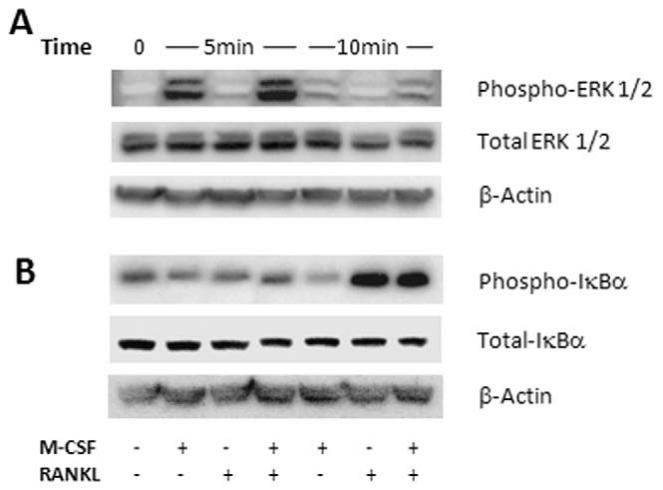
Activation of ERK1/2 and IκBα by RANKL and M-CSF in mature OCs. Mature OCs were serum starved for 18 h then acutely treated with RANKL (125 ng/mL) or M-CSF (25 ng/mL) alone, or in combination. Cytoplasmic protein extracts were harvested and subjected to SDS-PAGE followed by western analysis utilising antibodies against total and phosphorylated ERK1/2 and IκBα. A. Augmentation of phospho-ERK1/2 in the presence of RANKL and M-CSF. B. M-CSF has no effect on phospho-IκBα; representative data from 2 independent experiments.

Since MEK/ERK pathways are known to be involved in both RANK and c-fms signalling [22]–[24], we hypothesised that they may be involved in the observed effect of M-CSF on RANKL-induced resorbing activity. Furthermore, c-fos induction by ERK is well characterised [25], [26] and in the current study we have shown that c-fos is activated by M-CSF. In serum-starved OCs we found no detectable levels of constitutively phosphorylated ERK1/2. Treatment with M-CSF activated phosphorylation, with a peak increase of 477% at 5 min, dissipating to 70% at 10 min (Fig. 4A). Treatment with RANKL produced only a weak phosphorylation of ERK1/2 at 5 min, in comparison to M-CSF, with negligible levels detected at 10 min. Co-treatment with RANKL and M-CSF increased ERK1/2 phosphorylation at 5 min (44% increase) and 10 min (+19%), compared to M-CSF alone. Levels of total ERK1/2 were unchanged over the time-course investigated.

M-CSF alone decreased phosphorylated IκBα by 42% at 10 min, whereas RANKL exposure stimulated phosphorylation by 278%. There was no additional effect in combination with M-CSF. Total IκBα was unchanged over the time-course (Fig. 4B).

### Blockade of MEK/ERK signalling inhibits M-CSF-induced stimulation of resorption

**Figure 5:**
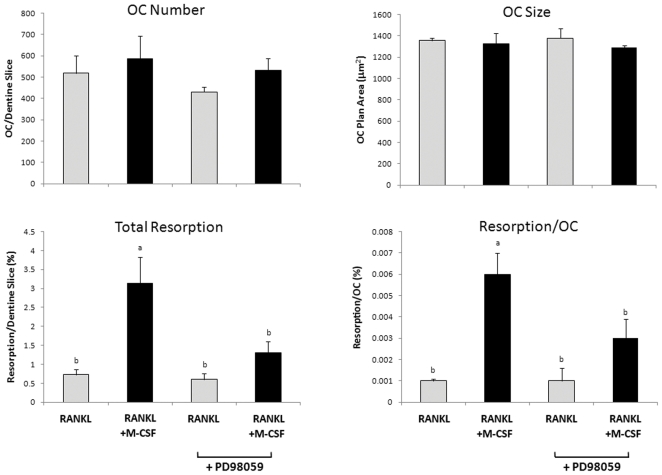
MEK inhibitor PD98059 blocks M-CSF induced stimulation of bone resorption by harvested OCs. Mature OCs where cultured for 72 h in the presence of RANKL (125 ng/mL) ± M-CSF (25 ng/mL) ± MEK inhibitor PD98059 (5 µM); representative data from 4 independent experiments. Results expressed as mean ± SEM (n = 6 dentine slices/group). Groups with different annotations are significantly different; p = 0.001; Oneway ANOVA; Fishers multiple comparison test.

The results above indicated that ERK1/2 phosphorylation was enhanced by co-treatment with M-CSF and RANKL, suggesting that MEK/ERK signalling may be involved in M-CSF enhancement of RANKL-induced resorption activity. To test this possibility, we examined the effects of PD98059, a specific inhibitor of the ERK1/2 activator MEK-1, on this effect. Mature OCs cultured on dentine slices were pre-treated for 1 h with PD98059 (5 µM), or vehicle, prior to treatment with RANKL alone or together with M-CSF for 72 h. Pre-treatment with PD98059 had no effect on OC survival, size or RANKL-induced resorption (Fig. 5). In cultures pre-treated with vehicle, treatment with RANKL and M-CSF produced a 330% increase in resorption compared to RANKL alone. This effect was attenuated in the presence of PD98059. In the experiment shown in Fig. 5, resorption in M-CSF group is not significantly different (ANOVA) to that of the RANKL-only control in the presence of PD98059. However, in 3 independent experiments PD98059 reduced the resorption-stimulating effect of M-CSF by 52.3±6.2% but resorption in the M-CSF group remained significantly greater than the control (p = 0.023, paired T-test).

## Discussion

Utilising a human, mature OC activation assay, we have demonstrated that M-CSF effectively activates OC resorption, in addition to its roles in survival and motility. It is established that RANKL rapidly activates resorption in isolated mouse OCs [27], increasing actin-ring formation within 30 minutes. Furthermore, treatment of mice with intravenous RANKL increases ionized calcium at 1 h, consistent with acute activation of mature OCs [27]. In our system, exposure to RANKL alone initiates, within 4 h, the formation of resorption pits that progressively enlarge with time, while the addition of M-CSF results in a rapid increase in the relative number of pits, which are not larger or longer, indicating a fundamental difference in the mechanism. The rapidity of this effect suggests that it is not due to an effect on OC differentiation but, rather, activation of mature cells. Pits progressively accumulate so the increased number could be due to an increase in the proportion of OCs activated to form pits, or an increase in the activation frequency among the same proportion of the OCs. However, since the presence of an actin ring indicates current OC activation [28], the observed increase in sealing zone number confirms that the increased pit number is due to a greater proportion of activated OCs.

Functional RANK is expressed on mature OCs and RANKL stimulates their activation [27], [29], [30]. Since resorption is RANKL-dependent, we surmised that augmentation of RANK signalling by M-CSF was a possible mechanism of its stimulation of OC activation. In OC precursors, a number of signalling cascades are known to be activated downstream of RANK leading to activation of NFκB, AP-1, p38, ERK, Src and NFAT (reviewed by Boyle et al. [12]). However, most of this knowledge is based on investigations done in rodent osteoclast generation models and the signalling pathways involved in the activation of mature OCs have not been well characterised. Likewise, signalling downstream of c-fms, the tyrosine kinase receptor of M-CSF is not well characterised in mature OCs although the important roles of Src kinase in resorption [31], [32], PI-3-kinase in motility [33] and PI-3-kinase as well as MEK 1 in survival [34] are well established. Therefore, we investigated the effects of acute treatment of OCs with RANKL on activation of NFκB, c-fos and NFATc1, and the modulation of this by M-CSF. In fully differentiated OCs under these experimental conditions, RANKL activation of NFκB is independent of M-CSF. However, RANKL activation of c-fos is dependent on the presence of M-CSF. NFATc1 appears to have no role in RANKL-induced activation of differentiated OCs. These results are consistent with the results of our resorption assays where RANKL initiates sub-maximal resorption activity independent of M-CSF while M-CSF augments RANKL-induced resorption, the former mediated by NFκB and the latter by c-fos.

Yao et al. [35] have previously shown that M-CSF treatment of mature mouse OCs results in increased c-fos expression and transcriptional activation [35]. The key role of c-fos in OC differentiation is well established [36], [37]. Our results indicate that in mature OCs c-fos activation is not essential for the initiation of resorption but that its activation by M-CSF greatly augments RANKL-induced activation of resorption. Moreover, although NFATc1 is critical in osteoclast differentiation it appears to play no part in resorption activation of fully differentiated OCs. Lee and Kim [26] have previously highlighted that existing data show that signalling pathways stimulated by RANKL are different in OC precursors and mature OCs [26].

Miyazaki et al. [24] used adenoviral gene transfer methods to activate and inhibit ERK and NFκB in semi-pure mouse OC cultures. Activation of ERK increased OC survival and inhibition of ERK decreased survival but neither had any effect on their bone-resorbing activity. In contrast, activation and inhibition of NFκB increased and decreased OC resorbing activity, respectively, but had no effect on survival [24]. We have shown that in fully differentiated human OCs, functional MEK/ERK pathways are not essential for the pro-survival and pro-resorption effects of RANKL but these pathways are involved in M-CSF augmentation of RANKL-induced resorption activation. The involvement of ERK is consistent with the involvement of c-fos activation that we have demonstrated. The results do not exclude the possibility that other MAPK parallel cascades, such as JNK and p38 are also involved [25].

Our novel finding that M-CSF regulates the rate of osteoclastic bone resorption *in vitro*, independent of OC formation, is supported by data from two murine *in vivo* studies. The first investigated the capacity of cell-surface M-CSF (csCSF-1) to restore *in vivo* M-CSF function in the CSF-1-deficient *osteopetrotic* (*Csf1^op^/Csf1^op^*) background [6]. Transgenic expression of csCSF-1 corrected the gross defects of these mice, including growth retardation and failure of tooth eruption. However, residual osteopetrosis and significantly delayed trabecular bone resorption in the subepiphyseal region of the long bones, along with incomplete correction of hematologic abnormalities were found. Complete restoration was achieved by transgenic expression of the full length CSF-1 gene, which encodes both circulating CSF-1 and some csCSF-1 [38]. Only partial correction of osteopetrosis in csCSF-1 transgenic mice was observed, even though there was an equivalent number and size of OCs to wild-type controls. This finding prompted the authors to conclude that while local csCSF-1 expression was sufficient to support osteoclastogenesis, the rate of bone resorption in these animals lacking circulating M-CSF was slower [6]. These investigators subsequently demonstrated [7] that the osteopetrosis phenotype of CSF-1- and CSF1R-deficient mice could be replicated by post-natal administration of a neutralising anti-CSF-1 antibody. The number of OCs was dramatically reduced at 15.5 but not at 36.5 or 64.5 days post-natal, and increased trabecular bone density remained. Very low to undetectable levels of circulating antibody were present in the older mice, suggesting that some M-CSF was present (although not measured in this study) but at sub-optimal levels for OC resorption to proceed [7].

In healthy individuals, serum M-CSF increases from approximately 12 ng/mL in the early twenties to 20 ng/mL in the eighties, corresponding to the concentration range where we observed *in vitro* a three- to four-fold increase in resorption [39]. In women, the serum bone resorption marker, C-telopeptide, increases in a near linear manner from age 21 to 71 years and bone mineral density declines substantially with ageing [40]. Thus, the possibility exists that increasing circulating M-CSF is involved in “normal” age-related bone loss. Furthermore, as we have previously reviewed, systemic and/or local M-CSF concentrations are increased in a variety of inflammatory and neoplastic conditions known to be associated with bone loss [11].

Karsdal et al. [41] pointed out that osteopetrotic mutations resulting in reduced or absent OCs are associated with decreased bone formation, whereas osteopetrotic mutations that result in increased numbers of non-resorbing OCs are associated with increased bone formation and suggested that nonresorbing osteoclasts provide anabolic signals for osteoblasts [41]. It has subsequently been shown that secreted products of OCs, spingosine-1-phosphate and bone morphogenic protein 6 stimulate migration and osteoblast differentiation of human mesenchymal stem cells [42].

We hypothesize that incomplete blockade of c-fms signalling, or downstream signalling pathways, may provide a potential means to attenuate bone loss. In this situation, OC differentiation will proceed but the proportion activated to resorb will be lower, resulting in reduced bone resorption yet maintenance of OC-derived anabolic signals to osteoblasts.

## References

[1] Hattersley G, Owens J, Flanagan AM, Chambers TJ (1991). Macrophage colony stimulating factor (M-CSF) is essential for osteoclast formation in vitro.. Biochem Biophys Res Commun.

[2] Marks SC, Lane PW (1976). Osteopetrosis, a new recessive skeletal mutation on chromosome 12 of the mouse.. J Hered.

[3] Tanaka S, Takahashi N, Udagawa N, Tamura T, Akatsu T (1993). Macrophage colony-stimulating factor is indispensable for both proliferation and differentiation of osteoclast progenitors.. J Clin Invest.

[4] Wiktor-Jedrzejczak W, Bartocci A, Ferrante AW, Ahmed-Ansari A, Sell KW (1990). Total absence of colony-stimulating factor 1 in the macrophage-deficient osteopetrotic (op/op) mouse.. Proc Natl Acad Sci U S A.

[5] Yoshida H, Hayashi S, Kunisada T, Ogawa M, Nishikawa S (1990). The murine mutation osteopetrosis is in the coding region of the macrophage colony stimulating factor gene.. Nature.

[6] Dai XM, Zong XH, Sylvestre V, Stanley ER (2004). Incomplete restoration of colony-stimulating factor 1 (CSF-1) function in CSF-1-deficient Csf1op/Csf1op mice by transgenic expression of cell surface CSF-1.. Blood.

[7] Wei S, Lightwood D, Ladyman H, Cross S, Neale H (2005). Modulation of CSF-1-regulated post-natal development with anti-CSF-1 antibody.. Immunobiology.

[8] Fuller K, Owens JM, Jagger CJ, Wilson A, Moss R (1993). Macrophage colony-stimulating factor stimulates survival and chemotactic behavior in isolated osteoclasts.. J Exp Med.

[9] Edwards M, Sarma U, Flanagan AM (1998). Macrophage colony-stimulating factor increases bone resorption by osteoclasts disaggregated from human fetal long bones.. Bone.

[10] Sarma U, Flanagan AM (1996). Macrophage colony-stimulating factor induces substantial osteoclast generation and bone resorption in human bone marrow cultures.. Blood.

[11] Hodge JM, Kirkland MA, Nicholson GC (2007). Multiple roles of M-CSF in human osteoclastogenesis.. J Cell Biochem.

[12] Boyle WJ, Simonet WS, Lacey DL (2003). Osteoclast differentiation and activation.. Nature.

[13] Bradley EW, Oursler MJ (2008). Osteoclast culture and resorption assays.. Methods Mol Biol.

[14] Bruzzaniti A, Baron R (2006). Molecular regulation of osteoclast activity.. Rev Endocr Metab Disord.

[15] Lee SK, Gardner AE, Kalinowski JF, Jastrzebski SL, Lorenzo JA (2006). RANKL-stimulated osteoclast-like cell formation in vitro is partially dependent on endogenous interleukin-1 production.. Bone.

[16] Lee SK, Lorenzo J (2006). Cytokines regulating osteoclast formation and function.. Curr Opin Rheumatol.

[17] Fuller K, Kirstein B, Chambers TJ (2006). Murine osteoclast formation and function: differential regulation by humoral agents.. Endocrinology.

[18] Lorenzo JA, Sousa SL, Alander C, Raisz LG, Dinarello CA (1987). Comparison of the bone-resorbing activity in the supernatants from phytohemagglutinin-stimulated human peripheral blood mononuclear cells with that of cytokines through the use of an antiserum to interleukin 1.. Endocrinology.

[19] Hodge JM, Kirkland MA, Aitken CJ, Waugh CM, Myers DE (2004). Osteoclastic potential of human CFU-GM: biphasic effect of GM-CSF.. J Bone Miner Res.

[20] Pavlos NJ, Xu J, Riedel D, Yeoh JS, Teitelbaum SL (2005). Rab3D regulates a novel vesicular trafficking pathway that is required for osteoclastic bone resorption.. Mol Cell Biol.

[21] Nicholson GC, Malakellis M, Collier FM, Cameron PU, Holloway WR (2000). Induction of osteoclasts from CD14-positive human peripheral blood mononuclear cells by receptor activator of nuclear factor kappaB ligand (RANKL).. Clin Sci (Lond).

[22] Dey A, She H, Kim L, Boruch A, Guris DL (2000). Colony-stimulating factor-1 receptor utilizes multiple signaling pathways to induce cyclin D2 expression.. Mol Biol Cell.

[23] Holmes SG, Still K, Buttle DJ, Bishop NJ, Grabowski PS (2004). Chemically modified tetracyclines act through multiple mechanisms directly on osteoclast precursors.. Bone.

[24] Miyazaki T, Katagiri H, Kanegae Y, Takayanagi H, Sawada Y (2000). Reciprocal role of ERK and NF-kappaB pathways in survival and activation of osteoclasts.. J Cell Biol.

[25] Cano E, Mahadevan LC (1995). Parallel signal processing among mammalian MAPKs.. Trends Biochem Sci.

[26] Lee ZH, Kim HH (2003). Signal transduction by receptor activator of nuclear factor kappa B in osteoclasts.. Biochem Biophys Res Commun.

[27] Lacey DL, Timms E, Tan HL, Kelley MJ, Dunstan CR (1998). Osteoprotegerin ligand is a cytokine that regulates osteoclast differentiation and activation.. Cell.

[28] Lakkakorpi PT, Vaananen HK (1991). Kinetics of the osteoclast cytoskeleton during the resorption cycle in vitro.. J Bone Miner Res.

[29] Fuller K, Wong B, Fox S, Choi Y, Chambers TJ (1998). TRANCE is necessary and sufficient for osteoblast-mediated activation of bone resorption in osteoclasts.. J Exp Med.

[30] Myers DE, Collier FM, Minkin C, Wang H, Holloway WR (1999). Expression of functional RANK on mature rat and human osteoclasts.. FEBS Lett.

[31] Miyazaki T, Sanjay A, Neff L, Tanaka S, Horne WC (2004). Src kinase activity is essential for osteoclast function.. J Biol Chem.

[32] Soriano P, Montgomery C, Geske R, Bradley A (1991). Targeted disruption of the c-src proto-oncogene leads to osteopetrosis in mice.. Cell.

[33] Grey A, Chen Y, Paliwal I, Carlberg K, Insogna K (2000). Evidence for a functional association between phosphatidylinositol 3-kinase and c-src in the spreading response of osteoclasts to colony-stimulating factor-1.. Endocrinology.

[34] Bradley EW, Ruan MM, Vrable A, Oursler MJ (2008). Pathway crosstalk between Ras/Raf and PI3K in promotion of M-CSF-induced MEK/ERK-mediated osteoclast survival.. J Cell Biochem.

[35] Yao GQ, Itokawa T, Paliwal I, Insogna K (2005). CSF-1 induces fos gene transcription and activates the transcription factor Elk-1 in mature osteoclasts.. Calcif Tissue Int.

[36] Grigoriadis AE, Wang ZQ, Cecchini MG, Hofstetter W, Felix R (1994). c-Fos: a key regulator of osteoclast-macrophage lineage determination and bone remodeling.. Science.

[37] Owens JM, Matsuo K, Nicholson GC, Wagner EF, Chambers TJ (1999). Fra-1 potentiates osteoclastic differentiation in osteoclast-macrophage precursor cell lines.. J Cell Physiol.

[38] Ryan GR, Dai XM, Dominguez MG, Tong W, Chuan F (2001). Rescue of the colony-stimulating factor 1 (CSF-1)-nullizygous mouse (Csf1(op)/Csf1(op)) phenotype with a CSF-1 transgene and identification of sites of local CSF-1 synthesis.. Blood.

[39] Suehiro A, Imagawa T, Hosokawa H, Suehiro M, Ohe Y (1999). Age related elevation of serum macrophage colony stimulating factor (M-CSF) level.. Arch Gerontol Geriatr.

[40] Desai MP, Bhanuprakash KV, Khatkhatay MI, Donde UM (2007). Age-related changes in bone turnover markers and ovarian hormones in premenopausal and postmenopausal Indian women.. J Clin Lab Anal.

[41] Karsdal MA, Martin TJ, Bollerslev J, Christiansen C, Henriksen K (2007). Are nonresorbing osteoclasts sources of bone anabolic activity?. J Bone Miner Res.

[42] Pederson L, Ruan M, Westendorf JJ, Khosla S, Oursler MJ (2008). Regulation of bone formation by osteoclasts involves Wnt/BMP signaling and the chemokine sphingosine-1-phosphate.. Proc Natl Acad Sci U S A.

